# Health risk assessment of exposure to Polycyclic Aromatic Hydrocarbons in bread from Iranian markets: Application of monte carlo simulation approach

**DOI:** 10.1371/journal.pone.0341584

**Published:** 2026-02-23

**Authors:** Najmeh Khodadadi, Masoumeh Saghi, Sina Mashayekhi, Negin Bashirian, Fateme Asadi Touranlou, Seyedeh Belin Tavakoly Sany

**Affiliations:** 1 Department of Health, Safety, and Environment Management, School of Health, Mashhad University of Medical Sciences, Mashhad, Iran; 2 Social Determinants of Health Research Center, Mashhad University of Medical Sciences, Mashhad, Iran; 3 Department of Environmental Health Engineering, Faculty of Health, Mazandaran University of Medical Sciences, Sari, Iran; 4 Student Research Committee, Department of Health, Safety, and Environment Management, School of Health, Mashhad University of Medical Sciences, Mashhad, Iran; 5 Division of Food Safety and Hygiene, Department of Environmental Health Engineering, School of Public Health, Tehran University of Medical Sciences, Tehran, Iran; The Islamic University, IRAQ

## Abstract

**Background:**

Polycyclic aromatic hydrocarbons (PAHs) are persistent environmental contaminants formed during high-temperature processing of food and are associated with both carcinogenic and non-carcinogenic health effects.

**Methods:**

A total of 96 bread samples (Lavash, Sangak, and Barbari) were collected from industrial and traditional bakeries across eight municipal zones of Mashhad, Iran. Sixteen priority PAHs were quantified by GC-MS after microwave-assisted saponification and DLLME clean-up. Toxic equivalent concentrations (BaPeq), daily dietary exposure, incremental lifetime cancer risk (ILCR), and Monte Carlo probabilistic modelling (100,000 iterations) were used for risk characterisation.

**Results:**

Mean Σ₁₆PAHs were significantly higher in traditional breads (68.63 ± 4.57 µg/kg) than industrial breads (39.87 ± 3.68 µg/kg; p < 0.001), with Lavash showing the highest levels (66.58 ± 6.02 µg/kg) and Sangak the lowest (40.95 ± 1.63 µg/kg). Naphthalene dominated light PAHs; fluoranthene and pyrene were predominant heavy PAHs. Mean BaP concentrations (0.05–0.10 µg/kg) were below the EU limit of 1 µg/kg. Mean TEQ was 0.18 µg/kg, with BaP contributing ~58%. Probabilistic assessment showed acceptable to low carcinogenic risk, slightly higher for traditional baking. Sensitivity analysis identified BaP as the primary driver. Compared globally, PAH levels in these Iranian breads indicate an acceptable risk, though continued monitoring and regulation are needed.

**Conclusion:**

Current PAH exposure via bread in Mashhad is generally acceptable, but traditional baking markedly increases contamination. Transition to indirect-heating ovens, improved ventilation, and regular monitoring are recommended to minimise exposure.

## 1. Introduction

Food safety is among the most pressing issues facing human societies today. Many infectious diseases that once threatened human health have been controlled or eradicated with advancements in science and knowledge [1,2]. The rising prevalence of non-communicable diseases (such as cancer, diabetes, and...) poses a significant concern for societies worldwide. Preventing these diseases is a top priority for health institutions worldwide. Adopting a healthy diet is one of the key factors that can positively influence the prevention of these diseases [3,4].

Bread is one of the most widely consumed foods across various cultures, and it is a staple food in Iran, where the average annual per capita consumption is a notably high 160 kg per year [5]. This significant consumption highlights the urgent need for regular monitoring of chemical contamination in bread, especially those with carcinogenic effects that pose serious health risks to consumers [6].

Polycyclic aromatic hydrocarbons (PAHs) are a group of persistent organic pollutants (POPs) classified as priority pollutants by the Agency for Toxic Substances and Disease Registry (ATSDR), the United States Environmental Protection Agency (EPA), and the European Union (EU). These compounds are characterized by the presence of two or more fused aromatic rings [2,6]. PAHs mainly arise from incomplete combustion and pyrolysis of organic matter. Anthropogenic sources include wood, coal, oil, tobacco, fossil-fuel combustion, vehicle exhaust, and sewage sludge, while natural sources comprise forest fires and volcanic eruptions [7,8]. These compounds pose a significant risk to human health as they are recognized to be carcinogenic [6,9]. Sixteen PAHs are classified as priority contaminants by the EPA. Humans are mainly exposed to PAHs via contaminated food, inhaled air, tobacco smoke, and cooking fumes [2,6]. Additionally, cooking at high temperatures increases the deposition of PAHs onto the surfaces of food [1,10]. The toxicity of PAHs varies based on several factors, including the type of exposure, concentration, duration of exposure, and the specific composition of the PAH mixture [3,6]. These compounds can have serious toxic effects on human health and are associated with various types of cancer, including skin, lung, pancreatic, esophageal, bladder, colon, and breast cancer in females [11]. PAHs can negatively impact reproductive health by affecting both sperm quality and quantity. They can disrupt sex hormones and harm the hormonal system. Additionally, PAHs may influence fetal development in pregnant women and have been linked to learning disabilities in children [6,12]. Currently, no regulations exist regarding the permissible concentration of PAH_16_ in bread. Nonetheless, the EU has stated that the concentrations of benzo[a]pyrene (BaP) and the total of PAH_4_ which includes chrysene (CHR), benzo[b]fluoranthene (B[b]F), BaP, and benzo[a]anthracene (B[a]A) should not exceed 1 ng/g in processed baby food and cereal-based products [13]. Additionally, the estimated average daily intake of BaP is approximately 4 ng/kg/day [14,15].

Studies indicate that cereal-based foods significantly contribute to human exposure to PAHs. Bread can be a health risk if it contains harmful levels of PAHs [2,15].

Given the absence of prior studies on PAHs in bread in the city of Mashhad and the significant role of bread in the Iranian diet, the primary aim of this study is to assess the health risks associated with PAHs exposure from bread consumption among Iranian consumers in Mashhad. This includes evaluating both cancer and non-cancer risks. This study addresses a critical regional data gap by providing the first comprehensive assessment of PAH levels and associated health risks in bread from Mashhad. The primary novelty of this research lies in the application of the Monte Carlo simulation approach (a probabilistic risk modeling technique) to provide a statistically robust estimation of dietary exposure and carcinogenic risk, thereby moving beyond the limitations of conventional deterministic risk assessment. This work provides essential, regional data to inform public health policy, enhance food safety, and promote safer baking practices in Iran.

## 2. Methods

### 2.1. Sample size and sample collection

In this study, the sample size was determined using a factorial design. We considered eight municipal areas in Mashhad, encompassing two cooking methods (industrial and traditional), three types of bread (Lavash, Sangak, and Berberi), and two repetitions. As a result, the total sample size was 96. The city of Mashhad was divided into eight regions based on municipal zoning. In each area, we selected six bakeries using a simple random sampling method, including three traditional and three industrial bakeries. For each type of bread—Sangak, Lavash, and Berberi—we chose two bakeries. From each bakery, we collected bread samples, totaling two samples per bakery, to analyze the concentration of 16 PAHs and assess their associated risk levels.

### 2.2. Chemicals and reagents

The polycyclic aromatic hydrocarbons was examined in this research included naphthalene (NAP), acenaphthene (ACE), acenaphthylene (ACY), phenanthrene (PHE), 2-bromophthalene (PBN-2), fluorene (FLR), fluoranthene (FLT), anthracene (ANT), pyrene (PYR), CHR, B[b]F, B[a]P, B[a]A, dibenz[a,h]anthracene (DB[ah]A), indene[1,2,3-cd] pyrene (I[c]P) and benzo[g,h,I]pyrylene (B[ghi]P). All individual PAH standard stock solutions (purity ≥98%) were supplied by Supelco (Bellefonte, PA, USA) at a concentration of 2000 µg/mL in dichloromethane. Biphenyl (purity ≥99%), used as the internal reference standard, was also obtained from Supelco (Bellefonte, PA, USA). Analytical-grade sodium chloride (purity ≥99.5%) and potassium hydroxide (purity ≥85%) were purchased from Merck (Darmstadt, Germany), along with HPLC-grade solvents (acetone, methanol, and tetrachloroethylene; purity ≥99.9%). Zinc acetate dihydrate (purity ≥98%) and potassium hexacyanoferrate(II) trihydrate (purity ≥99%) were obtained from Panreac (Barcelona, Spain). Hydrochloric acid (37%, analytical grade) and ethanol (purity ≥99.8%) were sourced from Merck (Darmstadt, Germany).

All stock solutions were supplied individually by Supelco (Bellefonte, PA) at a concentration of 2000 µg mL^-1^ in dichloromethane. Biphenyl served as the reference standard. Analytical-grade sodium chloride and potassium hydroxide, as well as HPLC-grade solvents, were purchased from Merck (Darmstadt, Germany). Zinc acetate and potassium hexacyanoferrate were obtained from Panreac (Belgium).

### 2.3. Extraction and analysis

For the analysis, a standard solution consisting of the 16 PAHs was prepared at a concentration of 100 μg/mL in methanol. Calibration standards ranging from 1 to 200 µg/mL were prepared through serial dilution of this standard solution.

PAHs were extracted and purified from samples of bread. Initially, 0.5 grams of bread powder were extracted using ethanol in a DeLonghi MW 602 microwave oven. This process involved adding a potassium hydroxide solution at a concentration of 2 mol/L and heating for 1.5 minutes. After the extraction, the resulting liquid extract was centrifuged for 5 minutes at 4,000 rpm to separate the liquid phase. The pH of the liquid was adjusted to 6.5 by adding hydrochloric acid (HCl) solution.

The purification and removal of interfering substances involved the preparation of two solutions. Solution 1 (Carrez I) was created by dissolving 10.6 g of potassium hexacyanoferrate in 100 ml of distilled water. Solution 2 (Carrez II) was prepared by mixing 21.9 g of zinc acetate with 3 ml of acetic acid, and then adjusting the total volume to 100 ml with distilled water. Both solutions were stored in a refrigerator at 4 °C before use.

Once the pH was adjusted, 1 milliliter each of Carrez I and Carrez II solutions was added to the sample. The mixture was stirred for one minute to precipitate the soluble carbohydrates. Finally, the mixture was centrifuged again at 4,000 rpm for an additional 5 minutes to separate the precipitates from the liquid.

The collected aqueous phase was used in the subsequent step of Dispersive Liquid-Liquid Microextraction (DLLME) [2]. Initially, 1.5 grams of sodium chloride were added to the sample and thoroughly mixed. A DLLME solution was then prepared, consisting of 300 µL of acetone, 80 µL of tetrachloroethylene, and 5 µL of biphenyl, which served as an internal standard at a concentration of 100 ng/mL. The solution was quickly introduced into the sample using a 500 µL syringe and gently mixed through vortexing to create a turbid suspension. The suspension was then centrifuged at 4,000 rpm to separate the solid precipitate from the liquid phase. Then, a 2 mL portion of the precipitate was subsequently injected into a gas chromatography-mass spectrometry (GC-MS) system using a 10 μL Hamilton microsyringe for analysis.

For the analysis of PAHs, we used an Agilent 7890A gas chromatograph (Agilent Technologies in Palo Alto, CA). This setup was combined with a three-axis detector and a 5975C mass selective detector (MSD). The gas chromatography-mass spectrometry (GC-MS) setup employed an HP-5MS capillary column that is 30 meters in length, with a diameter of 0.25 millimeters and a film thickness of 0.25 micrometers. The operating conditions were: Helium gas (99.99% purity) as the carrier at a flow rate of 0.8 mL min^-1^. The injector temperature was set to 250 °C and the auxiliary temperature to 280 °C.

The oven temperature was initially set to 150 °C and maintained for 2 minutes. It was then gradually increased to 200 °C at a rate of 8 °C min^-1^, where it was held for 1 minute. Subsequently, the temperature was raised to 250 °C at a rate of 5 °C min^-1^ and held for another 1 minute. The temperature was increased to 290 °C at a rate of 20 °C min^-1^ and maintained at this level for 10 minutes. A 1 μL injection volume was used for the analysis.

### 2.4. Quality control and assurance

At the beginning, midpoint, and end of each sample batch, blank samples containing the internal standard (IS) and quality control (QC) samples were prepared and analyzed. Additionally, all bread samples were tested in triplicate, and their average values were used for quantification.

Limits of detection (LOD) and quantification (LOQ) were calculated based on signal-to-noise ratios of 3:1 and 10:1, respectively, from seven replicate analyses of matrix-matched blanks spiked at low concentrations. Values were converted to µg/kg using the sample weight (0.5 g) and final extract volume. To ensure the reliability and validity of the analytical method, both the recovery rate and the method’s accuracy and precision were evaluated. Control samples with concentrations of 0.075 mg/kg and 0.75 mg/kg were prepared and analyzed using gas chromatography. Recovery percentages were determined by referencing the calibration curve, confirming that the method’s performance meets analytical standards. Three separate extractions were performed at varying concentration levels, all within the linear response range of the gas chromatography instrument, to assess accuracy. The relative standard deviation was calculated as an indicator of precision.

### 2.5. Health risk assessment

#### 2.5.1. Non-carcinogenic health risk.

##### 2.5.1.1. Toxic equivalence factors

The carcinogenic risks posed by PAH mixtures are predominantly evaluated using the BaP equivalent concentration and toxic equivalence factors (TEFs) (Table 1). This approach is widely regarded as a reliable standard for assessing the carcinogenic potential of PAH compounds. In this study, a specific set of TEF values was applied to calculate the BaP equivalent (BaPeq), following the methodology outlined by Xia et al. [20]. The Toxic Equivalent Quotient (TEQ) translates the concentration of a complex mixture of PAHs into an equivalent concentration of the reference carcinogen, Benzo[a]pyrene (BaP). The health risks associated posed by PAHs are primarily assessed using the concentration of BaPeq TEFs (Table 1). This method is considered a valid standard for evaluating the carcinogenic potency of PAH compounds. In this study, a specific set of TEF values was utilized to calculate the BaPeq, and the amount of BaP equivalent concentration in food was determined using equation (1). A TEQ value below 1 suggests that the cumulative toxicity of the PAH mixture is below the benchmark level of concern set by BaP.

**Table 1:** Values of health risk assessment parameters related to PAHs in bread (adult population).

Parameters	Unit	Abbreviation	value	Reference
Bread Intake Rate	kg/d	IR	0.23	[16]
		(Iranian consumption)		
Exposure Frequency	Days/Year	EF	365	[17]
Exposure Duration	Year	ED	70	[17]
Body Weight	Kg	BW	70	[17]
Average Time	Days	AT	25550	[18]
Oral Cancer				
Slope Factor of BaP	mg/kg/d	CSF	1	[4,19]
Toxicity Equivalency Factors: Nap, Ace, BPN2, Acen, FLO, PHE, FLO, PYR		TEF	0.001	
Toxicity Equivalency Factors: ANT, CHR, BgP		TEF	0.01	
Toxicity Equivalency Factors: BaA, BbF, IP		TEF	0.1	
Toxicity Equivalency Factors: BaP, DhA		TEF	1	


$$TEQ=\sum_{i=\text{0}}^{n}c_{i}\times TEF$$ (1)

In this study, “C_i_” refers to the concentrations of PAHs found in various cereal and bread products, while “TEF” denotes the toxic equivalency factor for each PAH. If the measured concentration of a specific PAH is below the limit of detection (LOD), it is recorded as half of the LOD value. To assess the carcinogenic potential of the 16 PAHs, the sum of their corresponding BaPeq values was calculated for each PAH. The daily dietary exposure level (ED) to PAHs for each group was estimated using equation (2).


$$ED=\sum_{i=\text{1}}^{n}TEQ\times {IR}_{i}$$ (2)

In this text, TEQ represents the concentration of PAHs in a food item (measured in ng g^-1^), while IR_i_ indicates the daily intake of each food type (measured in g d^-1^) [18].

#### 2.5.2. Carcinogenic risk.

The lifetime cancer risk (ILCR) for different groups exposed to PAHs was calculated using the following equation. In equation 3, ILCR estimates the probability of an individual developing cancer over a lifetime due to dietary exposure to PAHs. Regulatory agencies often consider an ILCR below 1 × 10 ⁻ ⁶ as negligible, between 1 × 10 ⁻ ⁶ and 1 × 10 ⁻ ⁴ as indicating an acceptable to low risk that may require monitoring, and above 1 × 10 ⁻ ⁴ as suggesting a potential concern that warrants intervention. The carcinogenicity slope factor (CSF) for BaP concerning oral cancer is established at 7.3 mg kg^-1^ day^-1^, according to the USEPA Guide for Risk Assessment in Superfund [4,19]. Following USEPA Guidelines for Carcinogen Risk Assessment, ILCR levels are interpreted as: negligible (<1 × 10 ⁻ ⁶; no further action needed), acceptable (1 × 10 ⁻ ⁶ to 1 × 10 ⁻ ⁴; monitoring recommended), and unacceptable/high (>1 × 10 ⁻ ⁴; intervention required).


$$\text{ILCR}=ED\times EF\times E_{D}\times CSF\times \frac{CF}{BW}\times AT$$ (3)

The variables used in equations (2) and (3) are defined in Table 1 [4,19].

### 2.6. Uncertainty and sensitivity analysis

In traditional risk assessment methods. The risk is usually estimated and presented as a single value. This single value gives quantitative insights into the level of uncertainty and variability linked to the estimated risk. To gain more precise insights into the risk or hazard index, the USEPA has introduced the Monte Carlo simulation method. Monte Carlo simulation utilizes mathematical statistics and probability theory to create an uncertainty model through random sampling and to establish a probability distribution for each input variable. In this study, we applied Monte Carlo simulation as a probabilistic method to reduce uncertainties. We used Crystal Ball software (version 11.1.34190) to conduct 10,000 iterations with a 95% confidence interval for the Monte Carlo modeling. The simulated model incorporated the 97.5th percentile of the hazard index and the carcinogenic risk as the primary health risk criteria. Probability distributions for input parameters were assigned based on empirical data and literature. PAHs concentrations (C) followed a lognormal distribution to account for their typically skewed nature in environmental samples. Bread consumption rate (IR) was modeled as a lognormal distribution, reflecting variability in dietary habits. Exposure frequency (EF) and exposure duration (ED) were assigned uniform distributions due to their consistent ranges across populations, while body weight (BW) followed a normal distribution based on the natural variability in human populations. Averaging time (AT) was modeled as a uniform distribution. These distributions were selected to reflect realistic variability and uncertainty in exposure parameters, as recommended by EPA guidelines and previous studies [21,22]. Additionally, Crystal Ball software enables the calculation of sensitivity analysis, which was employed in this study. We aim to evaluate the significance of each variable in the risk assessment.

**Fig 1:**
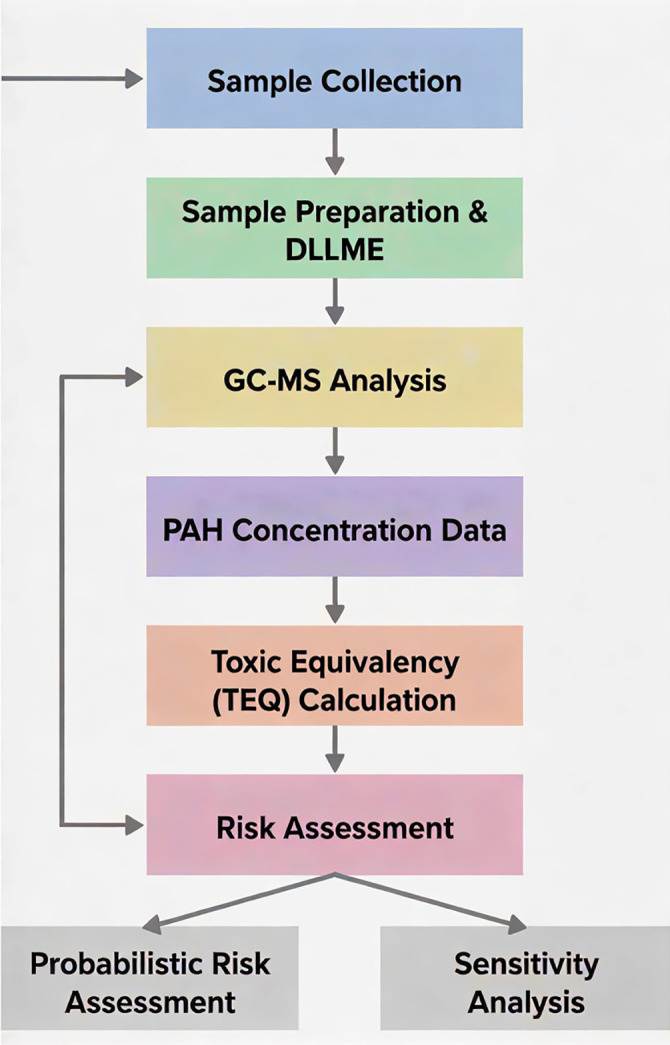
Overall workflow for the polycyclic aromatic hydrocarbon (PAH) health risk assessment.

Fig 1 illustrates the workﬂow diagram of this study.

### 2.7. Statistical analysis

SPSS statistical software was used to analyze the data. The Kolmogorov-Smirnov test assessed the normality of the dataset. To compare contaminant concentrations in bread, one-way ANOVA was applied to normally distributed data, while the Kruskal-Wallis test was used for non-normally distributed data. Descriptive statistics—including frequencies, means, and standard deviations—were employed to summarize the data, along with analysis of variance to compare changes in the variables.

### 2.8. Ethics approval

As this study involved the collection and analysis of commercially available food items and did not involve any human or animal subjects, the requirement for institutional review board approval and patient consent was not applicable.

## 3. Results and discussion

### 3.1. Analytical method evaluation

As shown in Table 2, this method demonstrates a broad linear range of 0.5 to 500 µg/kg, facilitating the detection of both trace and high concentrations of PAHs. The correlation coefficient (R^2^) ranges from 0.983 to 0.997, confirming acceptable linearity. Additionally, the method provides high sensitivity, with LOD from 0.045 to 0.083 µg/kg and quantification limits (LOQ) from 0.135 to 0.279 µg/kg. These features make the method highly effective for detecting trace levels of PAHs. The recovery rates for PAHs using this method range from 93.6% to 105.6%, demonstrating the extraction process’s high efficiency and minimal analytical loss. Furthermore, the method shows strong repeatability, with relative standard deviation (RSD%) values between 4.7% and 11.67%.

**Table 2:** Method validation and quality control parameters for measuring PAHs concentration (µg/kg) in bread samples.

PAHs	Linear range	LOD (µg/Kg)	LOQ (µg/Kg)	R^2^	Recoveries (%)	(RSDr) (%)
Naphthalene	0.10 −1000	0.055	0.165	0.984	98.2	8.9
Acenaphthylene	0.10 −1000	0.055	0.165	0.991	95.2	7.7
2-Bromonaphthalene	0.10 −1000	0.063	0.189	0.996	96.3	6.8
Acenaphthene	0.10 −1000	0.055	0.165	0.983	98.4	10.56
Fluorene	0.10 −1000	0.063	0.189	0.983	99.21	11.52
Phenanthrene	0.10 −1000	0.072	0.216	0.993	94.52	4.7
Anthracene	0.10 −1000	0.045	0.135	0.995	105.55	9.8
Fluoranthene	0.10 −1000	0.045	0.135	0.984	91.61	6.9
Pyrene	0.10 −1000	0.072	0.216	0.996	102.56	11.67
Benzo[a]anthracene	0.10 −1000	0.083	0.249	0.993	96.7	7.9
Chrysene	0.10 −1000	0.055	0.165	0.989	105.6	10.67
Benzo[b]fluoranthene	0.10 −1000	0.072	0.216	0.993	96.6	4.7
Benzo[a]pyrene	0.10 −1000	0.063	0.189	0.991	103.6	10.58
Indeno[1,2,3-cd] pyrene	0.10 −1000	0.045	0.135	0.997	91.6	6.9
Dibenz[a,h]anthracene	0.10 −1000	0.093	0.279	0.987	93.6	8.6
Benzo[g,h,i] perylene	0.10 −1000	0.063	0.189	0.993	102.6	9.87

LOD, limit of detection; LOQ, limit of quantification; R^2^, coefficient of determination; RSDR, relative standard deviation for reproducibility (inter-day precision, n = 6 replicates analyzed on three different days)

### 3.2. PAH_16_ concentrations in bread samples

Total Σ₁₆PAH concentrations were significantly influenced by baking method and bread type (detailed data in Supplementary S1 Table). Bread produced in traditional direct-fired ovens contained 68.63 ± 4.57 µg/kg, substantially higher than industrially baked bread (39.87 ± 3.68 µg/kg; p < 0.001). Among bread types, Lavash exhibited the highest mean concentration (66.58 ± 6.02 µg/kg), followed by Barbari (51.62 ± 2.06 µg/kg) and Sangak (40.95 ± 1.63 µg/kg). Light PAHs (2–3 rings) dominated the profile, comprising 89–94% of Σ₁₆PAHs in all categories, with naphthalene as the most abundant individual congener. Benzo[a]pyrene concentrations ranged from 0.05 to 0.10 µg/kg across all samples, well below the European Union maximum limit of 1 µg/kg. Three high-molecular-weight PAHs (indeno[1,2,3-cd]pyrene, dibenz[a,h]anthracene, and benzo[g,h,i]perylene) were below the limit of detection in every sample.

In Iran, the average concentration of PAH_16_ in different types of bread was examined across four studies conducted in Tehran, the capital of Iran [18,23,24]. A survey by Jahed Khaniki et al. (2021) showed that the average concentration of PAH_16_ ranged from 3.09 µg/kg in industrial bread to 31.36 µg/kg in Lavash bread, which is lower than the concentration reported in our study. The highest concentration was found in Lavash bread, at 31.36 µg/kg, while the lowest concentrations were 3.09 µg/kg in industrial bread and 4.12 µg/kg in Sangak bread, respectively [23]. Rostampour et al. (2017) found that the average level of PAHs in bread samples from Tehran was 15.55 µg/kg, which is lower than our results [25]. However, studies by Khalili et al. (2022) and Moradi et al. (2020) showed that the average concentration of PAH_16_ ranged from 64 to 176.3 µg/kg, which is higher than our findings. The lowest concentration was 64 µg/kg in industrial bread, and the highest was in Lavash and Taftoon breads, with values of 176.3 and 166.3 µg/kg, respectively [18,24].

To provide broader context, our findings were benchmarked against global data on PAH concentrations not only in bread but also in other cereal-based foods (e.g., spaghetti, flour, bran, and breakfast cereals), as these products share similar contamination pathways via raw materials and thermal processing. A recent systematic review and meta-analysis of 34 studies across 17 countries (1991–2023), encompassing 1057 bread samples, reported mean PAH16 concentrations in bread ranging from 1.06 µg/kg (Kuwait) to 374.8 µg/kg (Egypt) [4]. Similarly, a meta-analysis of 18 studies (1972–2021) on processed cereals worldwide (n = 639 articles screened) found mean PAH16 levels of 12.5 µg/kg in bread, 15.8 µg/kg in spaghetti, 22.3 µg/kg in flour, and 28.1 µg/kg in bran, with anthracene predominant in bread and naphthalene in bran [26]. These levels reflect regional variations driven by baking fuels and environmental pollution.

For instance, European breads (e.g., Spain: 1.29–11.70; Turkey: 5.72–11.74; UK: 2.06–2.29) show lower contamination due to stringent regulations and electric/gas ovens [4,27]. In contrast, higher levels were observed in Nigeria (2.26–105.24 µg/kg in bread; up to 248.3 µg/kg in cooked rice) and India (68.79–150.5 µg/kg in tandoori/tawa breads), often linked to wood/charcoal baking [2,4,28]. Kuwaiti toasted breads reached 92.14 µg/kg, while Egyptian samples hit extremes (30.05–374.8 µg/kg) from fossil fuel use [4,28]. Breakfast cereals and flour globally showed comparable or slightly higher means (e.g., 0.10–0.87 µg/kg PAH4 in Turkish cereals; 13.72 µg/kg pyrene in Nigerian breads) [4,26]. These comparisons underscore that while Mashhad’s traditional breads exceed industrial ones, they align with moderate-risk profiles in similar socio-economic contexts, emphasizing the need for fuel transitions to match European benchmarks.

Our findings indicated that the PAH_16_ concentration in different types of bread varied, and this difference in quantity probably resulted from baking methods and the type of fuel used in baking. PAHs are formed predominantly through pyrosynthesis when organic matter (starch, proteins, and lipids in dough) is exposed to temperatures >300 °C under limited oxygen conditions via Diels–Alder cyclisation, hydrogen-abstraction-acetylene-addition (HACA), and direct pyrolysis of fat dripping onto hot surfaces [12,29,30]. In traditional Iranian tanoor or stone ovens, the dough is in direct contact with flames or surfaces reaching temperatures of 400–600 °C, which strongly promotes these pathways. In contrast, indirect-heating industrial tunnel ovens maintain dough surface temperatures of only 200–250 °C and physically separate the combustion chamber, reducing PAH formation by 70–95% [31,32]. This mechanism explains the significantly higher contamination observed in Lavash (baked on hot metal plates/pebbles) compared with Sangak (shorter direct exposure on hot gravel). Traditional breads, especially lavash and those that employ fossil fuels or direct flames, showed the highest concentrations of PAH_16_, while industrial breads generally had lower levels. These results are in agreement with other studies that emphasize the influence of temperature, cooking methods, and type of fuel on the presence of PAHs in bread. They indicated that these discrepancies are directly linked to the type of fuel and the cooking method applied during the baking process [28,33]. Breads baked with fossil fuels, such as traditional breads, tend to accumulate more PAHs on their surfaces due to the heat generated [4,34]. Some studies compared the concentration of PAH_16_ in two types of bread: one cooked using traditional methods (fossil fuels and direct flames at a temperature of 300 °C) and another using an electric oven at 200 °C. Interestingly, a significant concentration of PAH_4_ and B[a]P (0.50 ± 0.25 µg/kg) was detected in bread prepared using traditional methods [26]. In contrast, PAHs were not observed in bread cooked in the electric oven. Likewise, the type of fuel has a notable impact on PAH concentrations. Bread baked using gas and electricity contains fewer PAHs [12]. Conversely, bread baked with fuels like solar fuels, wood, or diesel shows elevated concentrations of PAHs, especially B[a]P [4,34]. Other factors likely affecting PAH concentrations in different types of bread in Iran include the PAH levels in raw materials, water, bakery ingredients, and notably, wheat flour [2,12,28].

### 3.3. BaP, Light/High PAHs concentration in breads

B[a]P, classified as a Group 1 carcinogen by both the International Agency for Research on Cancer (IARC) and the Scientific Committee on Food (SCF) due to its established carcinogenic potential in humans [35–37], is subject to stringent regulatory limits. EU guidelines set the permissible limit for B[a]P in processed cereal-based foods at 1.0 µg/kg [35]. In this study, the average concentration of B[a]P in bread samples ranged from 0.05 ± 0.10 to 0.10 ± 0.08 µg/kg, which was under permissible limits (1.0 µg/kg) in all bread samples (Table 3).

**Table 3:** Average concentration of PAHs (µg/kg) in different locations in Mashhad city.

PAHs	location								
	1	2	3	4	5	6	7	8	Sig
Naphthalene	28.40 ± 4.03	28.17 ± 5.80	30.55 ± 8.38	30.43 ± 8.96	36.02 ± 9.96	31.28 ± 18.99	45.44 ± 26.48	41.51 ± 23.32	0.449
Acenaphthylene	0.43 ± 0.15	0.46 ± 0.41	0.49 ± 0.23	0.79 ± 0.91	0.43 ± 0.43	0.77 ± 0.52	0.68 ± 0.52	0.74 ± 0.93	0.605
2-Bromonaphthalene	4.44 ± 2.79	6.37 ± 5.33	6.51 ± 2.48	5.00 ± 3.88	5.43 ± 1.84	3.75 ± 2.51	6.47 ± 1.84	6.80 ± 2.11	0.153
Acenaphthene	3.06 ± 4.12	4.66 ± 5.08	3.95 ± 2.48	3.05 ± 3.23	2.82 ± 1.90	1.69 ± 1.88	3.96 ± 1.86	4.30 ± 2.11	0.207
Fluorene	1.69 ± 2.03	3.20 ± 3.46	2.08 ± 1.41	2.34 ± 2.99	2.07 ± 1.59	1.39 ± 1.38	3.00 ± 1.77	3.34 ± 2.00	0.325
Phenanthrene	1.62 ± 2.01	2.27 ± 2.41	1.59 ± 1.11	2.28 ± 2.96	1.96 ± 1.50	1.18 ± 1.17	2.42 ± 1.77	2.27 ± 1.65	0.723
Anthracene	1.52 ± 1.96	2.51 ± 2.41	1.62 ± 1.27	2.08 ± 2.83	1.37 ± 1.20	1.30 ± 1.34	1.97 ± 1.44	2.17 ± 1.74	0.676
Fluoranthene	0.94 ± 1.25	1.25 ± 1.20	1.00 ± 0.65	1.04 ± 1.41	0.56 ± 0.46	0.85 ± 0.72	1.73 ± 1.31	0.91 ± 0.75	0.396
Pyrene	1.24 ± 1.29	1.15 ± 1.05	1.07 ± 0.65	1.00 ± 1.33	0.57 ± 0.50	0.97 ± 0.72	1.94 ± 1.46	0.99 ± 0.82	0.393
Benzo[a]anthracene	0.34 ± 0.48	0.36 ± 0.37	0.35 ± 0.21	0.30 ± 0.46	0.19 ± 0.16	0.31 ± 0.25	0.64 ± 0.48	0.21 ± 0.32	0.125
Chrysene	0.65 ± 0.78	0.59 ± 0.85	0.39 ± 0.21	0.65 ± 1.20	0.29 ± 0.16	0.35 ± 0.25	0.68 ± 0.48	0.83 ± 1.09	0.451
Benzo[b]fluoranthene	0.14 ± 0.24	0.14 ± 0.26	0.08 ± 0.09	0.15 ± 0.27	0.06 ± 0.06	0.09 ± 0.08	0.19 ± 0.15	0.19 ± 0.22	0.379
Benzo[a]pyrene	0.04 ± 0.04	0.04 ± 0.05	0.03 ± 0.03	0.03 ± 0.05	0.05 ± 0.03	0.05 ± 0.05	0.12 ± 0.09	0.12 ± 0.14	0.065
Indeno[1,2,3-cd]pyrene	ND	ND	ND	ND	ND	ND	ND	ND	–
Dibenz[a,h]anthracene	ND	ND	ND	ND	ND	ND	ND	ND	–
Benzo[g,h,i]perylene	ND	ND	ND	ND	ND	ND	ND	ND	–
Total PAHs	44.58 ± 1.88	51.24 ± 2.68	49.77 ± 2.34	49.20 ± 2.93	51.86 ± 2.65	44.04 ± 4.85	69.29 ± 6.71	64.42 ± 5.94	–
L-PAHs	41.20 ± 2.75	47.67 ± 3.99	46.82 ± 3.53	46.00 ± 4.34	50.12 ± 4.01	41.39 ± 7.32	63.96 ± 10.11	61.15 ± 8.97	–
H-PAHs	3.38 ± 0.67	3.56 ± 0.62	2.95 ± 0.32	3.19 ± 0.78	1.74 ± 0.23	2.65 ± 0.36	5.33 ± 0.69	3.27 ± 0.53	–

LPAHs = light- polyaromatic hydrocarbons, HPAHs = heavy-polyaromatic hydrocarbons.

In Iran, the average concentration of B[a]P in bread was reviewed across eight studies [14,24,25,38–42], of which only three studies reported B[a]P concentrations exceeding the permissible limits set by EU standards, ranging from 1.0 to 2.11 µg/kg. High B[a]P concentrations were primarily found in bread samples baked using traditional methods involving fossil fuels and high-temperature cooking processes [12,40,42]. Additionally, the presence of PAHs in raw materials, such as flour, contributed to PAH contamination in bread samples [1,10]. However, the other five studies in Iran aligned with our findings, as B[a]P concentrations in bread samples were below the EU maximum level, ranging from non-detectable to 0.93 µg/kg. The lower B[a]P concentrations observed in these studies can be attributed to effective monitoring systems, strict food processing regulations, and the use of industrial baking methods with electronic and gas ovens [14,24,25,38–42].

Globally, a systematic review of 32 studies from 17 countries revealed mean B[a]P concentrations in bread ranging from non-detectable to 45.1 µg/kg [4]. Our findings align with those from European countries such as France [43], Poland [44], and Spain [45], where B[a]P concentrations in bread samples were below permissible limits. This consistency can be attributed to rigorous regulations, stringent control measures, and precise detection methodologies implemented to manage chemical contamination in food products. Conversely, studies conducted in Italy [12], China [1], Egypt [28,46], Kuwait [27], and India [10] reported B[a]P concentrations exceeding established standards, highlighting regional variations in food safety practices and regulatory enforcement.

The significantly higher PAH concentrations in traditional breads are a direct consequence of the baking methodology and fuel type. Traditional Iranian tanoor ovens often operate on a direct-heating principle, where the dough is placed on hot surfaces (e.g., pebbles for Sangak, hot metal for Lavash) that are in direct contact with the flame or are heated to extreme temperatures (400–600 °C). This environment is ideal for PAH formation through three primary pathways: (1) the pyrolysis of organic matter (starch, proteins, lipids) in the dough at temperatures exceeding 300 °C; (2) the Diels–Alder cyclisation and hydrogen-abstraction-acetylene-addition (HACA) mechanisms in the gas phase; and (3) the direct flare-up and deposition of smoke from the incomplete combustion of solid fuels like wood or diesel, which are still used in some traditional bakeries [29,47]. In contrast, industrial tunnel ovens utilize indirect heating, physically separating the combustion chamber from the baking chamber. This design maintains a lower and more uniform dough surface temperature (200–250 °C) and prevents the direct deposition of combustion products, thereby reducing PAH formation by 70–95% [31,32]. The higher levels in Lavash compared to Sangak can be attributed to its thinner structure and larger surface area exposed to the hot surface, potentially leading to more intense and rapid heating that favors PAH generation.

In this study, the mean concentrations of Light Polycyclic Aromatic Hydrocarbons (L-PAHs) varied significantly across different bread types, with Barbari exhibiting 47.82 ± 3.06 µg/kg, Lavash 62.79 ± 9.07 µg/kg, and Sangak 38.76 ± 2.42 µg/kg. Among the L-PAHs, NAP was consistently the most abundant congener across all tested samples, while B[a]P and B[b]F were observed at the lowest concentrations. The predominance of Naphthalene is consistent with other studies highlighting its significant contribution to overall PAH_16_ contamination and its utility as a common marker for PAH pollution [4,34]. The persistence and volatility of NAP facilitate its easy transfer and vaporization into bread during the baking process [28,46].

Conversely, in our study, the mean concentrations of Heavy Polycyclic Aromatic Hydrocarbons (H-PAHs) were recorded as 3.80 ± 0.54 µg/kg for Barbari, 3.79 ± 0.66 µg/kg for Lavash, and 2.18 ± 0.43 µg/kg for Sangak. Among the assessed carcinogenic/mutagenic PAHs, FLT and PYR were the most abundant H-PAHs. In contrast, I[c]P, DB[ah]A, and B[ghi]P were not detected in any of the analyzed samples, indicating their minimal presence in the tested bread types. Pyrene was identified as the predominant compound among the individual H-PAHs. A comparative analysis with previous research, such as the study by Jahed Khaniki et al. (2021) on bread samples in Tehran, revealed lower concentrations of H-PAHs (0.18–0.08 µg/kg) and L-PAHs (0.2–6.34 µg/kg) in their findings [23], which were lower than our results. These breads are baked in industrial ways, such as electronic and gas ovens, which generally lead to lower PAH formation compared to traditional methods.

An evaluation of PAH levels across eight urban areas of Mashhad (as presented in Table 3) indicated no statistically significant differences in PAH concentrations among these regions (p-value > 0.05). This suggests that geographical location within Mashhad does not exert a major influence on the overall PAH burden in bread. The observed homogeneity may be attributed to several factors: (1) a centralized or common supply chain for wheat flour and raw materials serving bakeries across the city, leading to a uniform initial contaminant load; (2) relatively similar ambient air quality and urban pollution profiles across Mashhad’s zones, resulting in comparable environmental deposition on ingredients and in bakery environments; and (3) the widespread prevalence of similar traditional and industrial baking practices (and fuel types) throughout the city, which appears to be a more dominant factor determining PAH levels than micro-location. However, Region 7 exhibited marginally higher concentrations of PAHs. This subtle variation could potentially be attributed to localized factors such as specific traditional baking practices, the types of fuel employed, or the efficiency of ventilation systems within the bakeries in that particular region, warranting further targeted investigation.

**Fig 2:**
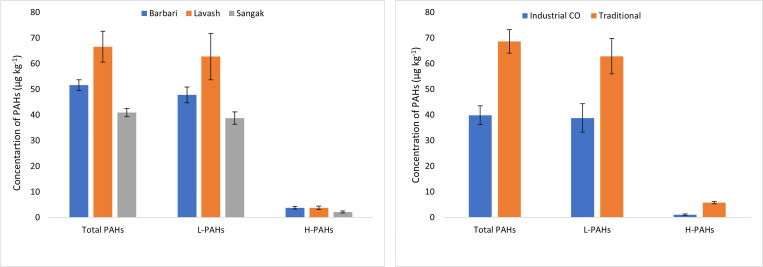
Mean concentrations of the 16 priority polycyclic aromatic hydrocarbons (PAHs) (µg/kg) in Lavash, Sangak, and Barbari breads produced by traditional and industrial baking methods in Mashhad, Iran.

Fig 2 shows the average concentration of 16 priority polycyclic aromatic hydrocarbons (PAHs) (µg/kg) in lavash, Sangak, and Berberi breads produced by traditional and industrial baking methods in Mashhad, Iran.

### 3.4. Health risk assessment

In this study, we carried out a comprehensive health risk assessment by measuring the Toxic Equivalent Concentrations (TEQs) for PAHs in various bread samples, as shown in Table 4. Importantly, all measured TEQ values for Lavash, Sangak, and Barbari breads, regardless of whether they were produced using industrial or traditional baking methods, remained below this established threshold. This suggests that, based on our current data, consuming these commonly available Iranian breads does not pose a significant health risk related to PAH exposure.

**Table 4:** Health risk assessment of PAHs content in bread consumed in Mashhad city.

Bread samples		TEQs	CR
**Type of bread**	Lavash	0.21 ± 0.16	7 × 10^−5^ ± 4.4 × 10^−5^
	Sangak	0.14 ± 0.14	5 × 10^−5^ ± 3.6 × 10^−5^
	Barbari	0.20 ± 0.15	7 × 10^−5^ ± 3.9 × 10^−5^
**Type of cook**	Industrial	0.08 ± 0.07	2.9 × 10^−5^ ± 1.5 × 10^−5^
	Traditional	0.30 ± 0.14	9 × 10^−5^ ± 3.8 × 10^−5^

TEQs: this is the toxic equivalent quantity of pollutant relative to B[a]P; CR: Carcinogenic risk based on the cancer slope factor of B[a]P; Mean±SD

**Fig 3:**
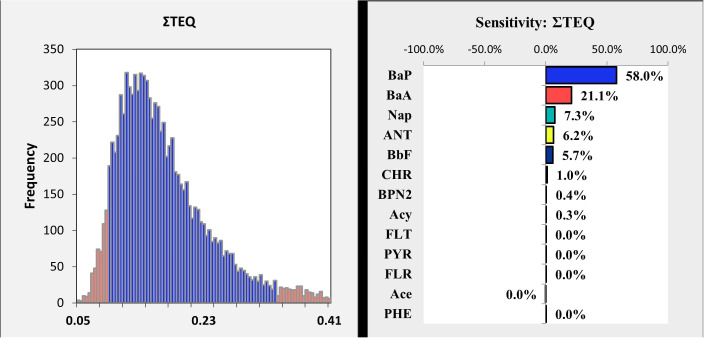
Left: The cumulative distribution of the TEQ health index of potentially toxic elements through bread consumption. Right: the effect of different variables on the TEQ health index caused by bread consumption.

In this study, a probabilistic health risk assessment was performed using Crystal Ball software, employing a Monte Carlo simulation with 100,000 iterations to determine the health risk index (Fig 3). The average risk index was calculated to be 0.18, with a 95% confidence interval from 0.09 to 0.34. Since this range is significantly below the acceptable threshold of TEQ < 1, both the average risk index and the confidence interval indicate an acceptable health risk, posing minimal concern for consumer health related to the consumption of the analyzed bread samples.

A sensitivity analysis, as depicted in the accompanying chart, identified B[a]P as the most significant contributor to the TEQ health index, accounting for approximately 58% of its total impact. B[a]A ranked as the second most influential PAH, contributing around 21.1%. Other notable contributors included NAP (7.3%), ANT (6.2%), and B[b]F (5.7%). CHR had a minimal impact, contributing only 1%, while compounds such as 2-Bromonaphthalene contributed negligibly (less than 1%) to the overall health risk index. The dominance of Benzo[a]pyrene (B[a]P), contributing over 50% to the total risk, was anticipated due to its high potency (TEF = 1). The substantial contribution of Benz[a]anthracene (B[a]A, ~ 18–20%) is particularly noteworthy. B[a]A is not only carcinogenic itself but also often acts as a direct precursor in the pyrosynthetic formation of B[a]P and other heavy PAHs during high-temperature combustion processes [48]. This shared formation pathway means that mitigation strategies designed to reduce B[a]P will inherently also reduce B[a]A. Therefore, focusing on interventions that disrupt the formation of these specific compounds—such as lowering baking temperatures below 300°C, using indirect heating to prevent direct flame contact, and ensuring complete combustion with cleaner fuels like natural gas—represents the most efficient approach to significantly reducing the overall carcinogenic risk associated with bread consumption.“

In contrast to our findings, other studies conducted in Iran have reported TEQ values ranging from 0.007 to 4.22 µg/kg, significantly exceeding the health risk threshold [12,40,42]. The disparity between our findings and these older studies can be attributed to several key factors related to temporal changes and methodological differences. The studies by Orecchio & Papuzza [12] and Mahmoudpour et al. (2018) [40] specifically analyzed bread baked using wood as a primary fuel. Over the past decade, there has been a gradual but significant shift in Iran, particularly in urban centers like Mashhad, towards cleaner fuels such as natural gas in both traditional and industrial settings, driven by municipal regulations and economic factors. Our sampling, conducted in 2023–2024, reflects this more modern fuel mix, which inherently produces lower PAH emissions compared to the wood-fired ovens prevalent during earlier study periods. Furthermore, the quality of wheat flour, a potential source of initial PAH contamination, has likely improved due to better agricultural practices, storage conditions, and milling technologies over time. Stricter controls on soil contamination and post-harvest handling could contribute to a lower initial PAH load in the raw materials used in contemporary bakeries. Additionally, analytical methodological advancements should be considered. Earlier studies often employed extraction and clean-up methods with potentially higher background interference or lower selectivity. The use of modern techniques in our work, such as optimized microwave-assisted extraction and dispersive liquid-liquid microextraction (DLLME) followed by high-sensitivity GC-MS, allows for more accurate quantification and might have resulted in lower, more specific measurements by better eliminating co-extracted compounds that could overestimate PAH levels in older methodologies. Therefore, while the older studies accurately reflected the high-risk scenario of their time, our current results likely represent a more updated and improved food safety profile, underscoring the positive impact of evolving practices and regulations. However, the persistence of a risk gradient between traditional and industrial methods in our data indicates that further progress is still possible and necessary.

However, the health risk indices observed in our study are consistent with findings from numerous countries worldwide, such as Romania, South Korea, Latvia, Nigeria, Ghana, Turkey, Spain, China, France, Poland, Saudi Arabia, Kuwait, and other European nations, where reported TEQ values remain well below the acceptable threshold [4,34,43–45]. In these international studies, TEQ values ranged from 0.001 µg/kg in Ghana to 0.899 µg/kg in Kuwait. On the other hand, the average TEQ values in bread samples from certain countries, including Italy [12], India [10], China [1], Egypt [28,46], and Kuwait [27], were notably above the acceptable threshold, ranging from 1.26 µg/kg in Kuwait to 149 µg/kg in Egypt. These bread types predominantly included Baguette, Toast, Tandoor, Tawa, Pita, Yontia, and Oily breads, suggesting a potential link between bread type and PAH contamination level [4,34].

### 3.5. Carcinogenic risk

The data in Table 4 shows that all measured CR values stayed below the standard index of 1 × 10 ⁻ ⁴. This indicates that, in this study, there is no significant carcinogenic risk associated with consuming the tested bread types or using the baking methods. Notably, traditional baking methods presented the highest carcinogenic risk, with an average CR value of 9 × 10⁻⁵.In contrast, industrial baking methods showed the lowest risk, with an average CR value of 2.9 × 10⁻⁵.

**Fig 4:**
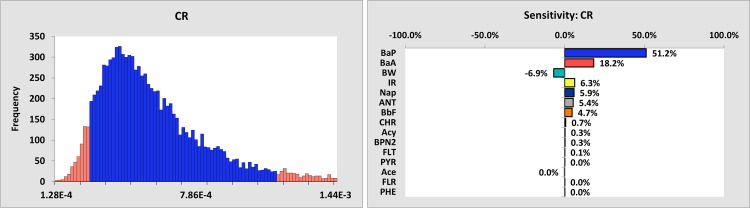
Left: The cumulative distribution of carcinogenic risk index of potentially toxic elements through bread consumption. Right: the effect of different variables on the carcinogenic risk index caused by bread consumption.

Using a probabilistic approach with Crystal Ball software and 100,000 iterations, the carcinogenic risk (CR) was assessed (Fig 4). The average CR was estimated at 6 × 10⁻⁵, with a 95% confidence interval ranging from 2.81 × 10⁻⁵ to 1.17 × 10⁻⁴. These values exceeded the acceptable limit in some samples, indicating a moderate carcinogenic risk. Sensitivity analysis revealed that Benzo[a]pyrene (B[a]P) and Benz[a]anthracene significantly influenced the carcinogenic risk index, accounting for 51.2% and 18.2%, respectively. Naphthalene (5.9%), Anthracene (5.4%), and Benzo[b]fluoranthene (4.7%) also had considerable effects. Body Weight (BW) had a negative impact (−6.9%), suggesting that increased weight reduces sensitivity, while the Ingestion Rate (IR) had a positive effect (6.3%) on carcinogenic risk. This result suggests that sub-populations with lower body weights, such as children, who also consume bread, would theoretically have a higher estimated daily exposure and, consequently, a higher carcinogenic risk compared to the average adult consumer, assuming a constant consumption rate. While our comprehensive probabilistic model accounts for this variability in BW, future studies should consider a dedicated, age-specific risk assessment for the child population to inform targeted public health guidance. Chrysene’s contribution was minimal (0.7%), and PBN had a negligible effect (0.3%).

A review of 34 studies measuring carcinogenic risk in bread samples revealed that 44.11% of studies from Iran, Turkey, South Korea, China, Poland, Spain, France, Latvia, Nigeria, and Ghana reported moderate CR levels (10⁻⁶ ≤ CR ≤ 10⁻⁴), aligning with our findings [4,34]. Conversely, 55.89% of studies found moderate to high carcinogenic risks (CR >10⁻⁴) linked to PAH-contaminated bread in Egypt, Iran, India, Italy, Saudi Arabia, and Turkey. The variation in CR across regions may result from differences in environmental contamination, bread processing methods, raw materials, and dietary habits [40,42]. While the mean ILCR (2.3 × 10⁻⁵) falls within the typical acceptable range (10⁻6 to 10⁻⁴), the 97.5th percentile ILCR (1.17 × 10⁻⁴) exceeds the conservative US EPA acceptable threshold of 10⁻^6^, demonstrating that high-end consumers face a potential lifetime cancer risk from bread consumption. This necessitates targeted risk management measures.

The present findings directly contribute to Sustainable Development Goals 3.4 and 3.9 (reduction of non-communicable diseases and illnesses from hazardous chemicals and pollution), SDG 7 (affordable and clean energy), SDG 11.6 (reduction of urban environmental impact), and SDG 12 (sustainable consumption and production) by demonstrating that replacing direct-fired traditional ovens with indirect-heating systems using natural gas/electricity markedly reduces carcinogenic PAH exposure in a staple food.

### 3.6. Limitations and future research

We acknowledge that this study is subject to certain limitations. Our health risk assessment focused exclusively on dietary exposure to PAHs through bread consumption, which is a major pathway in Iran. However, we did not account for non-dietary exposure pathways such as inhalation of polluted air, dermal contact, or exposure from other food sources (e.g., grilled meat, smoked foods), which could contribute to the overall body burden of PAHs. Therefore, the calculated Incremental Lifetime Cancer Risk (ILCR) should be interpreted as the risk specifically attributable to bread consumption. Future studies should aim to conduct a full exposure assessment incorporating all relevant environmental and dietary sources to provide a complete picture of total PAH-related health risks in the Iranian population.

## 4. Conclusion

This study provides a comprehensive health risk assessment of polycyclic aromatic hydrocarbons (PAHs) in commercially available breads from Mashhad, Iran. Our findings indicate that the concentrations of the 16 priority PAHs and the specific carcinogen benzo[a]pyrene (B[a]P) in all tested bread samples were below the EU regulatory limits. The calculated Toxic Equivalent Quotients (TEQs) and Carcinogenic Risk (CR) values suggest that the consumption of these breads generally poses an acceptable to low risk to the Iranian population.

However, several critical insights emerged with direct practical relevance. The significantly higher PAH levels in traditionally baked breads are clearly linked to the use of direct-heating methods and less controlled combustion conditions, which promote the pyrolysis of organic matter and the deposition of combustion products. Sensitivity analysis identified B[a]P as the most influential congener, contributing over 50% to the overall carcinogenic risk, highlighting it as a primary target for future mitigation efforts. While the current risk is deemed acceptable for the general population, the probabilistic assessment revealed that the upper confidence interval for CR (97.5th percentile of 1.17 × 10 ⁻ ⁴) approaches levels of concern, indicating a potential risk for high-consumption subgroups or under less optimal conditions.

These results align with Outcome 2.3 of the United Nations Sustainable Development Cooperation Framework (UNSDCF) for the Islamic Republic of Iran 2023–2027 [49], which aims to ensure that “people benefit from a cleaner and healthier environment with reduced exposure to pollutants and hazardous chemicals” by 2027. Transition to cleaner baking technologies, therefore, supports both national food-safety policy and Iran’s commitments under the UNSDCF [50].

To effectively reduce PAH formation in bread and minimize consumer exposure, the following evidence-based measures are strongly recommended:
1. Transitioning from Traditional Fuels and Methods: The most effective strategy is to systematically shift from traditional, direct-fire ovens and the use of high-PAH-producing fossil fuels (such as wood or diesel) to modern, indirect-heating ovens that use cleaner fuels (natural gas or electricity).
2. Optimizing Baking Conditions and Baker Education: Bakers, particularly in traditional settings, should be trained to avoid excessively high baking temperatures and prolonged baking times. Improving bakery ventilation can also help disperse combustion by-products away from the food.
3. Quality Control of Raw Materials: Implementing stringent quality control measures for raw ingredients, especially wheat flour, is crucial for minimizing the initial PAH load introduced before baking.
4. Stricter Regulatory Enforcement and Consumer Guidance: National food safety bodies should consider establishing and rigorously enforcing maximum permissible limits for Benzo[a]pyrene (B[a]P) and the sum of PAH4 in bread, aligning with international standards. Concurrently, clear guidance can help consumers make informed choices about dietary sources of PAHs.

## Supporting information

S1 Table Average concentration of PAHs (µg/kg) in different types of breads consumed in Mashhad city. (DOCX)

## References

[1] Li G, Wu S, Zeng J, Wang L, Yu W. Effect of frying and aluminium on the levels and migration of parent and oxygenated PAHs in a popular Chinese fried bread youtiao. Food Chem. 2016;209:123–30. doi: 10.1016/j.foodchem.2016.04.036 27173543

[2] Chawda S, Tarafdar A, Sinha A, Mishra BK. Profiling and health risk assessment of PAHs content in tandoori and tawa bread from India. Polycyclic Aromatic Compounds. 2020.

[3] Negoiță M, Mihai AL, Adascălului AC. Polycyclic Aromatic Hydrocarbons (PAHs) Determination in Cereals and Cereal-Based Products Using a Modified Quechers Method with Z-Sep⁺ Clean-Up and GC-MS/MS Quantification. Ms Quantification.

[4] Asadi Touranlou F, Hashemi M, Ghavami V, Tavakoly Sany SB. Concentration of polycyclic aromatic hydrocarbons (PAHs) in bread and health risk assessment across the globe: A systematic review and meta‐analysis. Compr Rev Food Sci Food Saf. 2024;23(5):e13411. 10.1111/1541-4337.13411 39245919

[5] Alami A, Banoorkar S, Rostamiyan T, Asadzadeh SN, Morteza MM. Quality assessment of traditional breads in Gonabad bakeries, Iran. J Res Health. 2014;4(3):835–41.

[6] Montano L, Baldini GM, Piscopo M, Liguori G, Lombardi R, Ricciardi M, et al. Polycyclic Aromatic Hydrocarbons (PAHs) in the Environment: Occupational Exposure, Health Risks and Fertility Implications. Toxics. 2025;13(3):151. doi: 10.3390/toxics13030151 40137477 PMC11946043

[7] Abdel-Shafy HI, Mansour MSM. A review on polycyclic aromatic hydrocarbons: Source, environmental impact, effect on human health and remediation. Egypt J Petrol. 2016;25(1):107–23. doi: 10.1016/j.ejpe.2015.03.011

[8] Khatri A, Kumar K, Thakur IS. Occurrence, fate, effects, risk assessment, biodegradation, and bio-transformation of polycyclic aromatic hydrocarbons (PAHs) and health hazards of their metabolites. Environmental Toxicants and Lifestyle Diseases. Springer. 2025. p. 55–81.

[9] Nsonwu-Anyanwu AC, Helal M, Khaked A, Eworo R, Usoro CAO, El-Sikaily A. Polycyclic aromatic hydrocarbons content of food, water and vegetables and associated cancer risk assessment in Southern Nigeria. PLoS One. 2024;19(7):e0306418. doi: 10.1371/journal.pone.0306418 39042616 PMC11265677

[10] Chawda S, Tarafdar A, Sinha A, Mishra BK. Profiling and Health Risk Assessment of PAHs Content in Tandoori and Tawa Bread from India. Polycycl Aromat Compound. 2017;40(1):21–32. doi: 10.1080/10406638.2017.1349679

[11] Amadou A, Praud D, Marques C, Noh H, Frenoy P, Vigneron A, et al. Dietary intake of polycyclic aromatic hydrocarbons (PAHs) and breast cancer risk: Evidence from the French E3N-Generations prospective cohort. Environ Int. 2025;200:109505. doi: 10.1016/j.envint.2025.109505 40373460

[12] Orecchio S, Papuzza V. Levels, fingerprint and daily intake of polycyclic aromatic hydrocarbons (PAHs) in bread baked using wood as fuel. J Hazard Mater. 2009;164(2–3):876–83. doi: 10.1016/j.jhazmat.2008.08.083 18842340

[13] European Commission E. Commission regulation (EU) no 835/2011 of 19 August 2011 amending regulation (EC) no 1881/2006 as regards maximum levels for polycyclic aromatic hydrocarbons in foodstuffs. Off J Eur Union. 2011;215(4):1–5.

[14] Peiravian F, Shakoori A, Moradi V, Salamzadeh J, Mahboubi A. Simultaneous Analysis of 10 Priority PAHs in Iranian Sangak Bread Samples by Developing a GC-MS Method. Iran J Pharm Res. 2021;20(2):433–40. doi: 10.22037/ijpr.2020.113074.14097 34567172 PMC8457723

[15] Abdulai PM, Bede-Ojimadu O, Onyena AP, Frazzoli C, Mogborukor NA, Ekhator OC, et al. Public Health Effects of Polycyclic Aromatic Hydrocarbons Exposure Through Air, Water, Soil, and Food in Ghana: Possible Economic Burden. Environ Health Insights. 2025;19:11786302251343767. 40548300 10.1177/11786302251343767 PMC12179457

[16] Kamalabadi M, Kamankesh M, Mohammadi A, Hadian Z, Ferdowsi R. Contamination and daily intake of polycyclic aromatic hydrocarbons in Iranian bread samples. Polycycl Aromat Compound. 2020.

[17] Asadi Touranlou F, Tavakoly Sany SB, Ghayour Mobarhan M, Khanzadi S, Afshari A, Hashemi M. Health Risk Assessment of Exposure to Heavy Metals in Wheat Flour from Iran Markets: Application of Monte Carlo Simulation Approach. Biol Trace Elem Res. 2025;203(4):2284–94. doi: 10.1007/s12011-024-04324-z 39083196

[18] Khalili F, Shariatifar N, Dehghani MH, Yaghmaeian K, Nodehi RN, Yaseri M, et al. The analysis and probabilistic health risk assessment of polycyclic aromatic hydrocarbons in cereal products. Environ Sci Pollut Res Int. 2022;29(21):31099–109. doi: 10.1007/s11356-021-17337-1 35000169

[19] IRIS E. Benzo [a] pyrene (BaP) CASRN 50-32-8| IRIS| US EPA, ORD. 2017.

[20] Xia Z, Duan X, Qiu W, Liu D, Wang B, Tao S, et al. Health risk assessment on dietary exposure to polycyclic aromatic hydrocarbons (PAHs) in Taiyuan, China. Sci Total Environ. 2010;408(22):5331–7. doi: 10.1016/j.scitotenv.2010.08.008 20800879

[21] Asadi Touranlou F, Tavakoly Sany SB, Ghayour Mobarhan M, Khanzadi S, Afshari A, Hashemi M. Health risk assessment of potentially toxic elements in bread from Iranian markets using Monte Carlo simulation. Sci Rep. 2025;15(1):38315. doi: 10.1038/s41598-025-22207-8 41184355 PMC12583719

[22] Asadi Touranlou F, Ghasemi S, Gholian Aval M, Hashemi M, Tavakoly Sany SB. Human health risk assessment of arsenic and potentially toxic elements exposure in bread and wheat flour in Northeast Iran. PLoS One. 2025;20(7):e0327652. 10.1371/journal.pone.0327652 PMC12286368 40700406

[23] Jahed Khaniki G, Ahmadi M, Ahmadabadi M, Shariatifar N, Ahmadkhaniha R, Rastkari N, et al. Assessment of polycyclic aromatic hydrocarbons (PAHs) in traditional breads consumed by people in Tehran city of Iran and the calculation of their daily intake. Int J Environ Anal Chem. 2021;103(11):2533–41. doi: 10.1080/03067319.2021.1895135

[24] Moradi V, Seyedain Ardabili SM, Shakoori A, Hoseyni SE. Development of a GC-MS Method for Determination of Various Polycyclic Aromatic Hydrocarbons in Iranian Traditional and Semi-industrial Taftoon Bread. Iran J Pharm Res. 2020;19(3):183–94. doi: 10.22037/ijpr.2019.15323.13018 33680021 PMC7757998

[25] Rostampour R, Kamalabadi M, Kamankesh M, Hadian Z, Jazaeri S, Mohammadi A, et al. An efficient, sensitive and fast microextraction method followed by gas chromatography-mass spectrometry for the determination of polycyclic aromatic hydrocarbons in bread samples. Anal Methods. 2017;9(44):6246–53. doi: 10.1039/c7ay02229h

[26] Einolghozati M, Talebi-Ghane E, Amirsadeghi S, Mehri F. Evaluation of polycyclic aromatic hydrocarbons (PAHs) in processed cereals: A meta-analysis study, systematic review, and health risk assessment. Heliyon. 2022;8(12):e12168. doi: 10.1016/j.heliyon.2022.e12168 36536913 PMC9758409

[27] Al-Rashdan A, Helaleh MIH, Nisar A, Ibtisam A, Al-Ballam Z. Determination of the levels of polycyclic aromatic hydrocarbons in toasted bread using gas chromatography mass spectrometry. Int J Anal Chem. 2010;2010:821216. doi: 10.1155/2010/821216 20862370 PMC2938452

[28] Ahmed MT, Abdel Hadi el-S, el-Samahy S, Youssof K. The influence of baking fuel on residues of polycyclic aromatic hydrocarbons and heavy metals in bread. J Hazard Mater. 2000;80(1–3):1–8. doi: 10.1016/s0304-3894(00)00300-9 11080564

[29] Rey-Salgueiro L, García-Falcón MS, Martínez-Carballo E, Simal-Gándara J. Effects of toasting procedures on the levels of polycyclic aromatic hydrocarbons in toasted bread. Food Chem. 2008;108(2):607–15. doi: 10.1016/j.foodchem.2007.11.026 26059139

[30] Lee J-G, Kim S-Y, Moon J-S, Kim S-H, Kang D-H, Yoon H-J. Effects of grilling procedures on levels of polycyclic aromatic hydrocarbons in grilled meats. Food Chem. 2016;199:632–8. doi: 10.1016/j.foodchem.2015.12.017 26776018

[31] Rose M, Holland J, Dowding A, Petch SRG, White S, Fernandes A, et al. Investigation into the formation of PAHs in foods prepared in the home to determine the effects of frying, grilling, barbecuing, toasting and roasting. Food Chem Toxicol. 2015;78:1–9. doi: 10.1016/j.fct.2014.12.018 25633345

[32] European Commission E. Commission regulation (EU) 2023/915 of 25 April 2023 on maximum levels for certain contaminants in food and repealing regulation (EC) no 1881/2006. Off J Eur Union. 2023;119:103–57.

[33] Ghasemi S, Hashemi M, Aval MG, Safarian M, Khanzadi S, Orooji A. Effect of baking methods types on residues of heavy metals in the different breads produced with wheat flour in Iran: A case study of Mashhad. J Chem Health Risks. 2022;12(1).

[34] Tavoosidana G, Abdolhosseini M, Mazaheri Y, Basaran B, Shavali-Gilani P, Sadighara P. The carcinogenic PAHs in breads, amount, analytical method and mitigation strategy, a systematic review study. BMC Public Health. 2024;24(1):1538. doi: 10.1186/s12889-024-18413-0 38849795 PMC11157925

[35] Palade LM, Negoiță M, Adascălului AC, Mihai AL. Polycyclic Aromatic Hydrocarbon Occurrence and Formation in Processed Meat, Edible Oils, and Cereal-Derived Products: A Review. Appl Sci. 2023;13(13):7877. doi: 10.3390/app13137877

[36] Sampaio GR, Guizellini GM, da Silva SA, de Almeida AP, Pinaffi-Langley ACC, Rogero MM, et al. Polycyclic Aromatic Hydrocarbons in Foods: Biological Effects, Legislation, Occurrence, Analytical Methods, and Strategies to Reduce Their Formation. Int J Mol Sci. 2021;22(11):6010. doi: 10.3390/ijms22116010 34199457 PMC8199595

[37] Kobets T, Smith BPC, Williams GM. Food-Borne Chemical Carcinogens and the Evidence for Human Cancer Risk. Foods. 2022;11(18):2828. doi: 10.3390/foods11182828 36140952 PMC9497933

[38] Khalili F, Shariatifar N, Dehghani MH, Yaghmaeian K, Nodehi RN, Yaseri M, et al. The analysis and probabilistic health risk assessment of polycyclic aromatic hydrocarbons in cereal products: with the chemometric approch. 2021. 10.1007/s11356-021-17337-1 35000169

[39] Kamalabadi M, Kamankesh M, Mohammadi A, Hadian Z, Ferdowsi R. Contamination and Daily Intake of Polycyclic Aromatic Hydrocarbons in Iranian Bread Samples. Polycycl Aromat Compd. 2019;40(4):1187–95. doi: 10.1080/10406638.2018.1534747

[40] Mahmoudpour M, Pilevar Z, Javanmardi F, Taram F, Mousavi M-M. PAHs in toasted bread: determination using microwave-assisted extraction and dispersive liquid–liquid microextraction followed by high-performance liquid chromatography. Anal Methods. 2018;10(20):2398–404. doi: 10.1039/c8ay00475g

[41] Eslamizad S, Kobarfard F, Javidnia K, Sadeghi R, Bayat M, Shahanipour S, et al. Determination of Benzo[a]pyrene in Traditional, Industrial and Semi- industrial Breads Using a Modified QuEChERS Extraction, Dispersive SPE and GC-MS and Estimation of its Dietary Intake. Iran J Pharm Res. 2016;15(Suppl):165–74. 28228814 PMC5242362

[42] Eslamizad S, Yazdanpanah H, Javidnia K, Sadeghi R, Bayat M, Shahabipour S, et al. Validation of an Analytical Method for Determination of Benzo[a]pyrene Bread using QuEChERS Method by GC-MS. Iran J Pharm Res. 2016;15(2):465–74. 27642317 PMC5018274

[43] Veyrand B, Sirot V, Durand S, Pollono C, Marchand P, Dervilly-Pinel G, et al. Human dietary exposure to polycyclic aromatic hydrocarbons: results of the second French Total Diet Study. Environ Int. 2013;54:11–7. doi: 10.1016/j.envint.2012.12.011 23376598

[44] Ciecierska M, Obiedziński MW. Polycyclic aromatic hydrocarbons in the bakery chain. Food Chem. 2013;141(1):1–9. doi: 10.1016/j.foodchem.2013.03.006 23768318

[45] Fasano E, Yebra-Pimentel I, Martínez-Carballo E, Simal-Gándara J. Profiling, distribution and levels of carcinogenic polycyclic aromatic hydrocarbons in traditional smoked plant and animal foods. Food Control. 2016;59:581–90. doi: 10.1016/j.foodcont.2015.06.036

[46] Atwa M. Determination of polycyclic aromatic of hydrocarbons (pahs) benzo [a] pyrene level in heat treated food from egyptian market by gc and hplc -fluoresences detection. J Agric Chem Biotechnol. 2015;6(6):137–53. doi: 10.21608/jacb.2015.44293

[47] Richter H, Howard JB. Formation of polycyclic aromatic hydrocarbons and their growth to soot—a review of chemical reaction pathways. Progr Energy Combust Sci. 2000;26(4–6):565–608. doi: 10.1016/s0360-1285(00)00009-5

[48] Bukowska B, Mokra, K, Michałowicz J. Benzo [a] pyrene—environmental occurrence, human exposure, and mechanisms of toxicity. Inter J Mol Sci. 2022;23(11):6348. 10.3390/ijms23116348 PMC9181839 35683027

[49] Sepehrnoush M. UNODC Country Partnership Programme in the Islamic Republic of Iran 2023-2026. 2023.

[50] Assembly UG. Transforming our world: the 2030 Agenda for Sustainable Development. 2015.

