# Supplementary material for: Health risk assessment of exposure to Polycyclic Aromatic Hydrocarbons in bread from Iranian markets: Application of monte carlo simulation approach
Source: PLoS One. 2026 Feb 23;21(2):e0341584. doi: 10.1371/journal.pone.0341584 (PMC12928422; doi:10.1371/journal.pone.0341584)
Supplement: S1 Table — (DOCX) [file pone.0341584.s001.docx]

Table S1. Average concentration of PAHs (µg/kg) in different types of breads consumed in Mashhad city.

| PAHs | Type of bread (Mean±SD) | | | | Type of cook (Mean±SD) | | |
| --- | --- | --- | --- | --- | --- | --- | --- |
|  | Barbari | Lavash | Sangak | Sig | Industrial CO | Traditional  co | P value* |
| Naphthalene (NAP) | 29.41±5.16 | 45.13±23.21 | 27.38±5.25 | <0.001 | 31.40±14.23 | 37.02±17.62 | <0.001 |
| Acenaphthylene (ACY) | 0.51±0.53 | 0.90±0.72 | 0.39±0.25 | <0.001 | 0.30±0.21 | 0.95±0.66 | <0.001 |
| 2-Bromonaphthalene (PBN-2) | 6.07±4.00 | 5.86±3.03 | 4.86±2.00 | 0.226 | 3.50±3.13 | 8.07±2.25 | <0.001 |
| Acenaphthene (ACE) | 4.08±3.17 | 3.86±3.61 | 2.37±2.01 | 0.144 | 1.38±1.52 | 5.86±2.64 | <0.001 |
| Fluorene (FLR) | 3.05±2.39 | 2.63±2.36 | 1.49±1.56 | 0.026 | 0.90±1.12 | 4.15±1.87 | <0.001 |
| Phenanthrene (PHE) | 2.38±1.96 | 2.27±2.18 | 1.20±1.21 | 0.052 | 0.70±0.83 | 3.43±1.71 | <0.001 |
| Anthracene (ANT) | 2.29±1.79 | 2.11±2.16 | 1.05±1.22 | 0.008 | 0.56±0.76 | 3.31±1.59 | <0.001 |
| Fluoranthene (FLT) | 1.32±1.11 | 1.18±1.14 | 0.61±0.72 | 0.008 | 0.33±0.48 | 1.86±0.91 | <0.001 |
| Pyrene (PYR) | 1.38±1.05 | 1.25±1.24 | 0.71±0.73 | 0.033 | 0.42±0.53 | 1.93±0.94 | <0.001 |
| Benzo[a]anthracene (B[a]A) | 0.43±0.38 | 0.40±0.42 | 0.18±0.26 | 0.020 | 0.10±0.18 | 0.62±0.34 | <0.001 |
| Chrysene (CHR) | 0.46±0.38 | 0.70±0.96 | 0.50±0.73 | 0.797 | 0.18±0.23 | 1.00±0.86 | <0.001 |
| Benzo[b]fluoranthene (B[b]F) | 0.12±0.12 | 0.18±0.25 | 0.10±0.16 | 0.488 | 0.02±0.06 | 0.26±0.21 | <0.001 |
| Benzo[a]pyrene (B[a]P) | 0.07±0.07 | 0.06±0.05 | 0.05±0.10 | 0.177 | 0.03±0.04 | 0.10±0.08 | <0.001 |
| Indeno[1,2,3-cd]pyrene (I[c]P) | ND | ND | ND |  | ND | ND | - |
| Dibenz[a,h]anthracene (DB[ah]A) | ND | ND | ND |  | ND | ND | - |
| Benzo[g,h,i]perylene (B[ghi]P) | ND | ND | ND |  | ND | ND | - |
| Total PAHs | 51.62±2.06 | 66.58±6.02 | 40.95±1.63 |  | 39.87±3.68 | 68.63±4.57 | <0.001 |
| L-PAHs | 47.82±3.06 | 62.79±9.07 | 38.76±2.42 |  | 38.79±5.56 | 62.86±6.88 | <0.001 |
| H-PAHs | 3.80±0.54 | 3.79±0.66 | 2.18±0.43 |  | 1.11±0.25 | 5.79±0.53 | <0.001 |

LPAHs = light- polyaromatic (NAP, ACY, PBN-2, ACE, FLR, PHE, and ANT/ hydrocarbons); HPAHs = heavy-polyaromatic hydrocarbons (FLT, PYR, B[a]A, CHR, B[a]P, B[b]F, I[c]P, DB[ah]A, and B[ghi]P.

ND = not detected

* Statistically significant differences between bread types (Kruskal–Wallis).
